# The Interplay between Obesity and Inflammation

**DOI:** 10.3390/life14070856

**Published:** 2024-07-08

**Authors:** Ilinca Savulescu-Fiedler, Razvan Mihalcea, Serban Dragosloveanu, Cristian Scheau, Radu Octavian Baz, Ana Caruntu, Andreea-Elena Scheau, Constantin Caruntu, Serban Nicolae Benea

**Affiliations:** 1 Department of Internal Medicine, The “Carol Davila” University of Medicine and Pharmacy, 050474 Bucharest, Romania; 2 Department of Internal Medicine and Cardiology, Coltea Clinical Hospital, 030167 Bucharest, Romania; 3 Department of Orthopaedics, “Foisor” Clinical Hospital of Orthopaedics, Traumatology and Osteoarticular TB, 021382 Bucharest, Romania; 4 Department of Orthopaedics and Traumatology, The “Carol Davila” University of Medicine and Pharmacy, 050474 Bucharest, Romania; 5 Department of Physiology, The “Carol Davila” University of Medicine and Pharmacy, 050474 Bucharest, Romaniacostin.caruntu@gmail.com (C.C.); 6 Department of Radiology and Medical Imaging, “Foisor” Clinical Hospital of Orthopaedics, Traumatology and Osteoarticular TB, 030167 Bucharest, Romania; 7 Clinical Laboratory of Radiology and Medical Imaging, “Sf. Apostol Andrei” County Emergency Hospital, 900591 Constanta, Romania; 8 Department of Radiology and Medical Imaging, Faculty of Medicine, “Ovidius” University, 900527 Constanta, Romania; 9 Department of Oral and Maxillofacial Surgery, “Carol Davila” Central Military Emergency Hospital, 010825 Bucharest, Romania; 10 Department of Oral and Maxillofacial Surgery, Faculty of Dental Medicine, “Titu Maiorescu” University, 031593 Bucharest, Romania; 11 Department of Radiology and Medical Imaging, Fundeni Clinical Institute, 022328 Bucharest, Romania; 12 Department of Dermatology, “Prof. N.C. Paulescu” National Institute of Diabetes, Nutrition and Metabolic Diseases, 011233 Bucharest, Romania; 13 Department of Infectious Diseases, The “Carol Davila” University of Medicine and Pharmacy, 050474 Bucharest, Romania; 14 “Prof. Dr. Matei Balș” National Institute for Infectious Diseases, 021105 Bucharest, Romania

**Keywords:** obesity, inflammation, adipocytes, adipose tissue, cytokines, adipokines, macrophages, weight loss

## Abstract

Obesity is an important condition affecting the quality of life of numerous patients and increasing their associated risk for multiple diseases, including tumors and immune-mediated disorders. Inflammation appears to play a major role in the development of obesity and represents a central point for the activity of cellular and humoral components in the adipose tissue. Macrophages play a key role as the main cellular component of the adipose tissue regulating the chronic inflammation and modulating the secretion and differentiation of various pro- and anti-inflammatory cytokines. Inflammation also involves a series of signaling pathways that might represent the focus for new therapies and interventions. Weight loss is essential in decreasing cardiometabolic risks and the degree of associated inflammation; however, the latter can persist for long after the excess weight is lost, and can involve changes in macrophage phenotypes that can ensure the metabolic adjustment. A clear understanding of the pathophysiological processes in the adipose tissue and the interplay between obesity and chronic inflammation can lead to a better understanding of the development of comorbidities and may ensure future targets for the treatment of obesity.

## 1. Introduction

Obesity is defined as an excessive accumulation of body fat and is caused by the disparity between energy intake and consumption, facilitated mainly by excessive caloric intake, sedentarism, mental disorders, or genetic factors [1]

Obesity increases the risk for metabolic disease, atherosclerosis, various malignant tumors, and a host of immune-mediated disorders due to a chronic, systemic, inflammatory response [2,3,4]. The inflammation is directly correlated with exceeding weight and is dominated by clear hallmarks such as an imbalance between pro- and anti-inflammatory processes, abnormal cytokine (CK) levels, and exaggerated production of acute-phase reactants, such as C-reactive protein (CRP) [5]. Numerous CKs, such as tumor necrosis factor alpha (TNF-α), IL (interleukin)-6, CRP, and IL-1β, are studied in the interplay between metabolic disease, inflammation, and carcinogenesis, but mediators of innate immunity, namely inflammasomes, are also regarded with increasing interest [6,7,8,9].

Chronic over-nutrition leads to systemic toxicity exerted by glucose and unoxidized long-chain fatty acids and can stimulate the synthesis of pro-inflammatory adipokines [10]. High-fat diet (HFD) feeding can cause imbalances in the adipose tissue (AT) environment and alter its anti-inflammatory state by recruiting pro-inflammatory immune cells; a chronic inflammatory state may be achieved, both local and systemic [11,12].

The inflammation induced by obesity is triggered in the white adipose tissue (WAT) and spreads to various other tissues [13]. The obesity-associated chronic inflammation of the AT ultimately leads to its dysfunction and involves fibrosis, reorganization of the extracellular matrix (ECM), hypoxia, and altered angiogenesis, among other mechanisms [14].

There is a causal relationship between excessive caloric intake and inflammation. Weight gain leads to an increased amount or volume of adipocytes and subsequent changes in the phenotype of WAT [7]. This phenotype shift is characterized by the development of inflamed adipocytes with altered function as well as the recruitment of immune cells that release pro-inflammatory CKs [11,15,16,17,18,19].

However, the exact trigger of chronic inflammation in obesity, as well as the pathways of CKs released from the AT leading to systemic inflammation, is not well understood.

Some important differences between classical and obesity-related inflammation are worth highlighting. In various experimental models and human trials, it was shown that chronic inflammation develops in obese individuals; however, the level of circulating CKs is unable to generate, by itself, a sufficient chemotactic force to draw in monocytes [20]. Moreover, AT inflammation induced by overnutrition is not associated with a significant increase in energy expenditure, compared to the traditional inflammation model, thereby explaining the coexistence of inflammation and weight gain [21].

The innate and adaptive immune systems play a significant role in sustaining the metabolic activity of the WAT [12]. A causative relationship between excessive feeding and the activation of the immune system in organs regulating energy metabolism was demonstrated in both human and animal studies [22,23].

This paper aims to highlight the interplay between obesity and chronic inflammation by focusing on the cellular and humoral components, allowing for a more precise and complete image on the inflammatory mechanisms at play in obesity. Moreover, we will present the comprehensive role of macrophages in the propagation and regulation of inflammation within the adipose tissue and present the signaling pathways that can be targeted in the treatment of high-risk obese individuals. Persistent inflammation after weight loss is a topic that is often overlooked in other studies and is explored in the current paper, focusing on the management of weight loss in obesity-associated inflammation.

## 2. Methodology

For documenting this review paper, we performed a search in electronic databases such as PubMed, Google Scholar, and Embase utilizing relevant search words including “obesity”, “inflammation”, “adipose”, “markers”, “immune”, “cytokines”, “signaling”, and “mechanism” in order to identify adequate papers on the topic. The grey literature was also screened, in order to obtain a comprehensive view of the topic and to ensure the inclusion of all relevant studies. Studies that were not in English or were not available in full-text were not included in this study.

## 3. Features of the Adipose Tissue

The AT is the largest fat-storing depot, an active immune and metabolic organ, and also the largest endocrine organ that secretes and releases adipokines, cytokines, and chemokines into the adjacent vascular network [24,25,26]. 

The AT comprises adipocytes, which make up less than 50% of the total cells, and the stromal vascular fraction [27]. The latter includes adipocytes not yet loaded with lipids (i.e., pre-adipocytes), cells belonging to the innate immune system (macrophages, neutrophils, eosinophils, mast cells), immune cells of the adaptive immune system (B cells, NKT, CD4, CD8 cells), vascular cells, and fibroblasts. Macrophages represent around 10% of a lean person’s AT but may increase up to 40% in obesity [28,29]. The innate response, mediated by neutrophils and macrophages, is rapid but non-specific. On the other hand, the adaptive response is mediated by T and B cells and is directed against a specific aggressor. The innate and adaptive responses reinforce each other; therefore, macrophages act as antigen-presenting cells for T cells, and, in turn, T cells release pro-inflammatory cytokines such as interferon-γ (IFN-γ), which further activates macrophages [30,31].

Adipocytes are important energy storages, that take up and release lipids within the adipose tissue, according to the signaled needs. Their number is determined during childhood and adolescence and remains relatively stable throughout an individual’s lifetime [32]. 

Adipocytes modulate the inflammatory response by secreting TNF-α and IL-6, adipokines with opposite effects on inflammation, acting as signaling molecules [33,34,35]. Also, a number of adipocyte-produced lipids such as palmitate and other unsaturated fatty acids interfere with inflammation, augmenting the pro-inflammatory signaling [36]. Adipocytes also secrete branched-chain fatty acid esters of hydroxyl fatty acids that inhibit inflammation and improve insulin secretion and sensitivity [37,38].

Adipose tissue (AT) can be classified through morphological considerations into brown adipose tissue (BAT) and WAT. AT mostly comprises WAT which is deposited mainly subcutaneously and periviscerally and represents a major factor in the development of cardiovascular conditions and complications [39]. BAT represents only a minor proportion of AT, is distributed in the cervical, thoracal, mediastinal, and abdominal areas, exerts anti-obesity and anti-diabetes roles, and is involved in thermogenesis [39,40,41,42,43].

The main feature of BAT is the inclusion of numerous lipid droplets and mitochondria, necessary for thermogenesis. Conversely, WAT includes larger adipocytes that contain fewer mitochondria and only one fat droplet, thus enabling the cells to store energy rather than expend it in exchange for heat [44,45].

According to its distribution, white adipose tissue can be classified into two main compartments: subcutaneous adipose tissue (SAT) and visceral adipose tissue (VAT), the latter comprising the adipose tissue deposited in the abdominal cavity, i.e., within the omentum, the mesentery, in the retroperitoneal space, and around the organs. There are several differences between SAT and VAT in terms of structure but also function [46]; furthermore, differences in lean and obese persons between these tissues are also noted. The volume of VAT is positively correlated with cardiovascular events and is a more significant risk factor than the BMI [47,48]. Perivascular adipose tissue surrounds blood vessels and hosts a variety of immune cells such as eosinophils and B cells, which were shown to be reduced in high-fat diets [49], and appear to be involved in vascular relaxation and atheroprotection [50].

As opposed to SAT, VAT has direct liver access, via the portal venous system [51], and is therefore involved in the hepatic production of C reactive protein [52]. VAT is richer in large adipocytes [53] and inflammatory cells [54]. VAT is the largest source of adiponectin [55], while SAT is the largest source of leptin [56]. 

Regarding the tissue structure, SAT includes small-size adipocytes with high avidity for FFA and triglyceride [57]. However, there are differences in the WAT structure in normal-weight and obese subjects. In lean persons, WAT contains mostly cells with regulatory and immunosuppressive actions: M2 macrophages, eosinophils, NKT, Th2 cells, and regulatory T cells. The macrophages are uniformly distributed within the AT in order to release anti-inflammatory CKs, assure the clearance of apoptotic adipocytes, and inhibit pre-adipocyte differentiation [29]. On the contrary, in the obese, the lipid storages of the WAT are increased and macrophages are increased in number and are inhomogeneously distributed, in clusters around apoptotic adipocytes, forming crown-like structures (CLSs) [36,58,59].

The VAT in obese persons contains more macrophages as compared to the SAT [60,61,62]. Moreover, VAT releases more inflammatory cells and does so more easily [54]. In obese persons, VAT inflammation is associated with hepatic and skeletal muscle ectopic lipoid deposits which may trigger insulin resistance [63]. The reported response to inflammation and apoptosis is more intense in VAT compared to SAT [64].

The capabilities of pre-adipocytes to differentiate into adipocytes are different between VAT and SAT, i.e., VAT expands via hypertrophy while SAT scales up mainly by hyperplasia [65]. The sensitivity to apoptosis is weaker in adipocytes from the SAT compared to the VAT [66]. Additionally, the uptake of FFA after meals is greater in VAT than in SAT [67,68]. 

**Figure 1:**
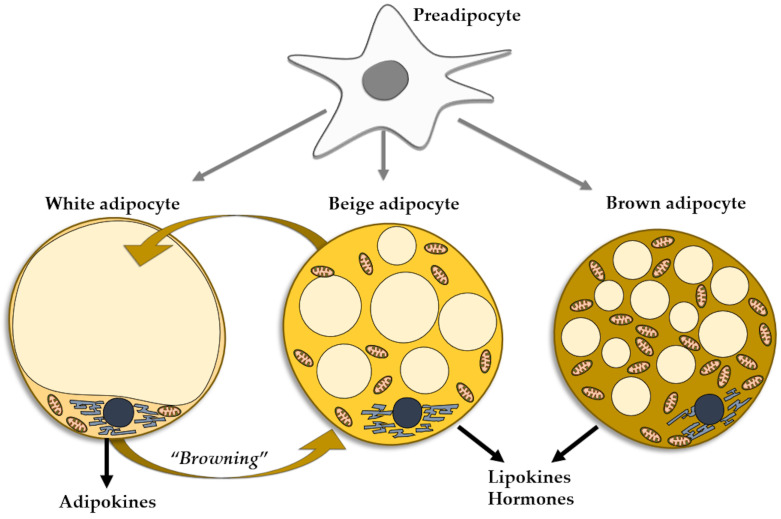
Types of adipocytes in the adipose tissue evolved from the common progenitor. Trans-differentiation between white and beige adipocytes.

The adipocytes found in the WAT can be mainly regarded as energy accumulators. However, the brown adipocytes of the BAT act as a heat source. A third type of adipocytes, named beige adipocytes, have the same origin as white adipocytes, but present the functional phenotype of brown cells [69]. Under certain conditions, mature white fat cells trans-differentiate into beige adipocytes, a process referred to as WAT browning, which entails the appearance of beige cells in WAT depots, more so in subcutaneous than in visceral WAT [6] (Figure 1). As mentioned, the phenomenon of WAT browning may occur in various conditions, including exercise [70], prolonged cold exposure [71], and exposure to capsaicin, resveratrol, fish oil, cinnamon, short fatty acids [72], and PPAR gamma agonists [73]. The browning of WAT may be considered as an environmental adaptation mechanism in adult life, similarly to the presence of brown adipocytes produced during intrauterine life [74]. Resembling brown adipocytes, beige adipocytes contain multilocular lipid droplets [75] and are rich in mitochondria, expressing uncoupling protein-1, a thermogenic protein [76]. Beige adipocytes secrete minute amounts of leptin and adiponectin, pertaining to the overall small mass of cells [77]. They are mainly heat producers, but also contribute to the improvement of glucose and lipid metabolisms and mounting evidence supports the thesis of beige adipocytes as protectors against obesity and insulin resistance. Similarly to BAT, beige tissue is considered as a therapeutic target for metabolic diseases, including obesity [77]. The reverse is also true, as in aging, obesity, and metabolic disorders the capacity of the WAT cells to induce browning of WAT is decreased [78] (Figure 1).

## 4. Molecular Mechanisms in Adipocyte Inflammation

Recent studies have shown that inflammation within the adipose tissue plays a paramount role in the development of metabolic syndrome and related complications. Moreover, chronic inflammation represents an intersection point of various molecular pathways impacting the immune system and potentially affecting the function of distant organs via cytokines [79]. Several signaling pathways may interfere with the expression of proteins regulating the cell cycle, apoptosis, or oxidative stress, leading to a higher risk of developing a subset of cancers [80,81,82].

Murine studies showed that FFA-mediated reactive oxygen species (ROS) generation induces endoplasmic reticulum (ER) stress [83]. Obese patients show a correspondence between the BMI and the level of ER markers, as well as between ROS generation and FFA levels [84,85]. FFAs directly stimulate toll-like receptor 4 (TLR4), promoting the inflammatory cascade [86]. It was also observed that tenascin C, an endogenous TLR4 activator, shows increased levels in the WAT of obese subjects [87]. Interestingly, the expression of anti-inflammatory counterregulatory TLR types such as TLR9 is also increased in obesity [88]. 

Elevated FFAs and ROS in the AT induce the release of pro-inflammatory adipokines, favor immune activation, and lead to chronic inflammation [89]. Increased oxidative stress was identified in obesity and its complications and it is triggered by the dysregulation of mitochondrial function [10]. The subsequent accumulation of ROS leads to nuclear factor-kappa B (NF-κB)-mediated apoptosis with associated inflammation due to increased production of inflammatory adipokines [90].

The senescence markers are elevated in hypertrophic VAT, and the secretion of senescent proinflammatory CKs from cells maintains and stimulates the inflammation within the adipose tissue and favors adipocyte apoptosis in obese persons [91,92,93].

Various signaling pathways may be activated or modulated by inflammation, and therefore represent potential new targets in the treatment of obesity-related metabolic dysfunctions.

The NF-κB signaling pathway plays a major role in the local and systemic inflammation induced by obesity [94,95,96]. The activation of the NF-kB inflammatory pathway leads to subsequent stimulated expression of pro-inflammatory CKs, which intensify the inflammatory processes [97,98].

In a second signaling pathway, the inflammasome multiprotein complexes that regulate the immune response following activation by myeloid cells are involved [99]. NLRP3 (NLR family pyrin domain containing 3) is a major representative of the immune metabolic response and is present in various tissues, including the CLSs of the AT [100,101]. The NLRP3 inflammasome requires pro-caspase 1 for activation and is associated with the release of IL-1β and IL-18 by AT macrophages [102,103,104,105]. Therefore, the NLRP3-IL-1β pathway is essential in the development of obesity-related complications and its downregulation was demonstrated to decrease the expression of pro-inflammatory CKs, inflammatory processes, and fibrosis within the AT [106,107]. Also, inhibition of NLRP3 in HFD showed a protective effect against liver steatosis and cardiovascular complications in murine studies [108]. On the other hand, blocking IL-1β via antagonists in type 2 DM or obesity showed a significant reduction in systemic inflammation [109,110,111].

Various proteins may be involved in the signaling processes regulating inflammation in obesity. Among these, galectin-3 is produced by macrophages, is upregulated in obesity, and shows a direct correlation with insulin resistance [112]. ATM-derived exosomes are also important factors in obesity-related insulin resistance and can shift their phenotype in lean versus obese individuals [113].

## 5. Obesity and Inflammation

The inflammatory response in inflammation includes cellular and humoral components. Early upstream markers for inflammation can be used in the detection and prevention of metabolic syndrome, in targeted therapies and anti-inflammatory treatments, in superior disease monitoring, as well as in public health programs that can identify at-risk obese individuals and facilitate early intervention [114,115]. Moreover, measuring certain adipokines can provide insight into the inflammatory status and can guide personalized interventions while some cytokines can be targeted in managing obesity-related metabolic disturbances [116].

### 5.1. Obesity and the Cellular Component of the Inflammatory Response

The inflammatory response in obesity is complex and features immune cells involved in both cellular and humoral immunity, to various degrees, and acting in different phases. 

#### 5.1.1. Macrophages

Macrophages are the main cellular component involved in the chronic inflammation of the AT in obese humans and animals [117,118]. 

Macrophages are responsible for clearing cellular waste originating from dead cells but they also contribute to angiogenesis as well as the remodeling of the ECM [119]. Macrophages are also essential in the expansion and remodeling of the AT during impaired adipogenesis [66].

The crosstalk between adipocytes and the AT macrophages triggers the process of systemic metabolic inflammation in obesity [120,121]. It was also shown that there is a relationship between adipose tissue macrophage (ATM) infiltration and resistance to insulin.

The central role that macrophages play in obesity is defined by their increase in number as well as the phenotype shift leading to several functional responses [122]. The number of macrophages can increase in obesity in various manners, e.g., through chemotaxis by capturing monocytes from the circulation, by increasing the proliferation of local macrophages, as well as through the stimulation of macrophage retention [23,29,123,124].

Monocytosis occurs during the migration of monocytes into the AT while the VAT releases monocyte chemoattractant protein-1 (MCP-1) and leukotriene B4 [20,125,126]. Osteopontin is also an important adipokine that attracts monocytes to the AT and contributes to macrophage proliferation and infiltration [127,128,129]. Additionally, calprotectin can contribute to the accumulation of monocytes within the inflammatory milieu following its release from the mature macrophages [129,130,131]. Hypoxia and adipocyte necrosis also contribute to macrophage recruitment [128].

In obese persons, a specific population of macrophages is encountered, comprising Ly6C+ monocyte-derived macrophages undergoing differentiation [132]. ATMs expressing the Ly6C monocyte marker were identified outside the CLSs and were shown to increase adipocyte differentiation [133].

In obesity, monocyte and macrophage infiltration enter a positive feedback, increasing their expression and ultimately leading to chronic inflammation within the WAT [7]. The proliferation of macrophages in the WAT occurs mainly within the CLSs and, under the stimulation of IL-4, leads to an increased selective expression of M2 macrophages [134,135].

The cellular features and tissue distribution of macrophages are distinct in obese compared to lean individuals. Obese mice have increased expression of pro-inflammatory cytokines such as TNF-α and inducible NO synthase (iNOs) while non-obese mice show increased expression of anti-inflammatory cytokines like IL-10 and arginase-1 [58]. The infiltration of ATM is further stimulated by the increase in NF-κB levels that extend the lifespan of macrophages, which subsequently contribute to maintaining the local inflammation [136].

After the death of adipocytes within the CLSs, macrophages accumulate in the periphery, a process mediated by the adipocytes releasing MCP-1 [137]. The accumulated macrophages then release TNF-α, stimulating a subsequent release of FFAs from the adipocytes [138]. FFAs will then bind to TLR4 on adipocytes and macrophages, resulting in the activation of NF-kB signaling and the release of IL-1β by the macrophages [139].

The population of macrophages in the AT comprises two main categories, M1 ATM which show pro-inflammatory features and are the majority in obese AT, and M2 ATM which exhibit anti-inflammatory effects and represent the main type in lean persons [58,140]. A third population of macrophages is described in obesity, with hybrid M1 and M2 characteristics, and plays a role in mitigating excessive lipids within the AT [141,142,143].

The M1 and M2 macrophages differ in two essential enzymes. M1 macrophages generate NO and ROS with the help of iNOS, while M2 macrophages use arginase to promote collagen production and deposition [144,145].

Another distinction between the two main types of macrophages is in ATP generation; M1 macrophages generate ATP quickly via the glycolysis pathway, while M2 macrophages use oxidative phosphorylation (OX PHOX) to obtain energy [146]. In lean mice, macrophages have low OX PHOX expression and a limited capacity for glycolysis; conversely, in obese mice, both types of pathways are overexpressed and highly effective [147]. Moreover, M1 macrophages produce lipids and pro-inflammatory lipid mediators while M2 macrophages contribute to lipid oxidation [148]. In activated M1 macrophages, eicosanoids are not the only pro-inflammatory components released; the synthesis of pro-inflammatory CKs such as TNF-α, IL-6, IL-12, and IL-23 is also increased, with a concomitant decrease in the anti-inflammatory CKs, such as IL-10 [149].

**Figure 2:**
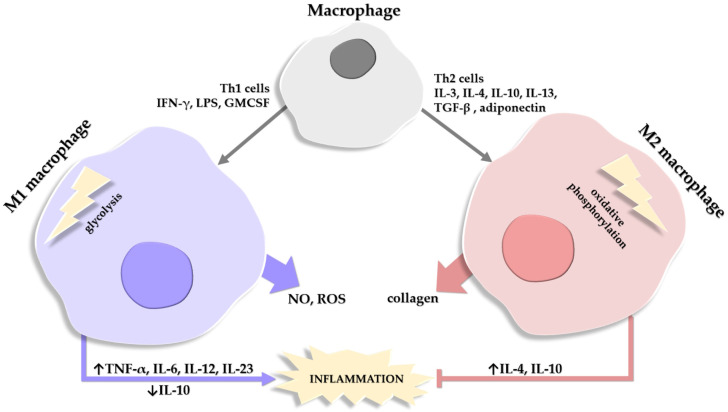
Schematic depiction of the factors involved in the differentiation of a macrophage into a type M1 or M2 macrophage. Macrophages release substances that degrade (NO, nitric oxide; ROS, reactive oxygen species) or help repair (collagen) the extracellular matrix. Roles of M1 and M2 macrophages in the balance of stimulating (→)/inhibiting (˫) inflammation are also depicted, alongside the cytokines involved in the process, where their expression/release is either stimulated (↑) or inhibited (↓).

An important mention is that the pro-inflammatory phenotype M1 in obesity is different from the phenotype of M1 macrophages in acute inflammatory reactions, in that M1 ATMs express lower levels of pro-inflammatory CKs like TNF-α and IL-6 compared to the classical acute inflammatory M1 response [143,150]. Additionally, a synchronous activation of the endogenous anti-inflammatory cascade is observed in obesity, with increased levels of IL-10, for example, which leads to the inhibition of macrophage activation [151]. Also, in obese persons, the distinct M1-like types of macrophages upregulate the expression of genes that encode proteins involved in lipid metabolism [150,152,153] (Figure 2).

CD9 is a cell surface marker expressed by various cells, including macrophages, and its expression can be regulated in inflammation and macrophage activation [154].

According to the CD9 expression, we can differentiate CD9+ ATMs, which are increased in obesity and are mostly derived from the bone marrow, and CD9- ATMs, which are responsible for angiogenesis [133,141]. CD9+ ATMs found within the CLSs show increased expression of pro-inflammatory mediators and CKs, as well as genes related to lipid metabolism and lysosomal pathways [20].

Macrophage polarization is one of the steps of the inflammatory reaction in obesity and is crucial for the secretion of pro-inflammatory mediators such as TNF-α, IL-6, and MCP-1 [155,156]. 

The expression of M1 and M2 markers can influence the activation and phenotype expression of the ATM [141]. The variability of phenotype expression is also known as phenotypic plasticity and allows for the adaptation to changes in the microenvironment (i.e., polarization); polarization towards the M1 type occurs through CKs produced by Th1 cells, while polarization towards the M2 type occurs via Th2 cells [128]. Most M1-like polarized ATMs derive from circulating monocytes and the transition is regulated by dietary or hydrolyzed bisaturated fatty acids via a TLR4-dependent mechanism [22,157,158,159,160]. The triglyceride-containing obese adipocytes are responsible for inducing an oxidative environment by stimulating ATMs to produce TNF-α, which subsequently increases the fatty acid release [10,136]. M1 macrophages resulting from the conversion of anti-inflammatory M2 macrophages further amplify the plethora of molecular events observed in obesity [161].

#### 5.1.2. Other Cellular Components

Neutrophils represent the largest fraction of the circulating leukocytes and their infiltration and subsequent activation within the adipose tissue has recently been linked to obesity-associated inflammation [162]. Murine models have been used to study the interaction between neutrophils, cytokines, and other cells, including macrophages, in the development of obesity-related complications in HFD [163]. Neutrophils are infrequent cells in the AT of lean mice [164,165]. However, in obesity, neutrophils are the first immune cells in the AT recruited from peripheral circulation [128]. The migration and activation of neutrophils are stimulated by the lipids accumulated within adipocytes, as well as leptin and various pro-inflammatory factors including TNF-α [128,166,167,168]. 

Murine studies showed that macrophage recruitment is decreased in neutrophil activity impairment demonstrated by the decreased expression of neutrophil enzymes [164,169]. Moreover, in obesity, neutrophil dysfunction leads to resistance to insulin and inflammation within the AT [170].

The infiltration of the VAT by neutrophils after HFD occurs well before the onset of insulin resistance or even weight gain resulting in macrophage recruitment [171]. Murine studies show that neutrophil infiltration occurs at 3 days after the HFD initiation and disappears after one month [171]. In humans suffering from obesity, however, the definitive role of neutrophils in AT inflammation is uncertain.

Eosinophils are key factors in obesity-associated inflammation; they play a physiological role in the regulation of metabolic hemostasis [172]. Eosinophils release high levels of IL-3, IL-4, IL-10, and TGF-β, leading to M2 polarization of ATMs, favoring the anti-inflammatory effects [173].

B cells are upregulated in obesity [174]. In murine studies on obesity, B cells accumulate in the AT and increase the recruitment of neutrophils and monocytes through the chemokines released within the microenvironment [165]. Furthermore, B cells promote the AT inflammation and the recruitment and activation of T cells [165,174,175], while also presenting antigens to T cells, releasing pro-inflammatory CKs and therefore contributing to the development of the inflammatory process [176,177,178].

T cells represent the second most common immune cell type after macrophages and are a population of cells significantly increased in obese mice undergoing HFD [152,175]. In particular, CD3+ T cells stimulate macrophage chemotaxis and differentiation [179]. The levels of CD3+CD4+ Th1 cells are higher in obese individuals and they release IFN-γ, stimulating inflammation, while the levels of CD3+CD4+ Th2 cells are lower, and they release IL-4 with anti-inflammatory effects [175,180,181].

Mast cells are found in increased numbers in the WAT of obese individuals [182]. Through degranulation, they release pro-inflammatory substances and facilitate the macrophage infiltration of the AT [182,183,184]. Within the WAT, the activity of mast cells is influenced by IFN-γ and IL-6 [182]. 

Regarding the cellular component, the WAT tends to have a higher concentration of pro-inflammatory immune cells such as M1 macrophages, neutrophils, and mast cells, as well as B and T cells, modulating metabolic inflammation and IR, while BAT presents more anti-inflammatory immune cells such as M2 macrophages, eosinophils, and innate lymphoid cells pertaining to its thermogenic and anti-inflammatory roles [7,163,185,186,187].

### 5.2. Obesity and Cytokines in the AT Inflammation

The CKs released in the AT have various effects, mainly pro-inflammatory ones. The amount of anti-inflammatory CKs appears to decrease with weight gain; therefore, the pro-inflammatory CKs become dominant. 

The levels of TNF-α, IL-1β, and IL-6 are elevated in obesity. However, their concentrations are far below the limits for exhibiting their biological effects, with the exception of IL-6 [188,189,190,191,192]. Therefore, a deeper and more complex analysis of the processes and interactions of the CKs is required for a better understanding of the inflammatory balance in obesity.

TNF-α plays a major role in adipocyte apoptosis as well as in cellular signaling in obesity-induced inflammation. The production of TNF-α occurs mainly in macrophages, as well as adipocytes, neutrophils, and other immune and supportive cells. After its release, TNF-α subsequently favors the production of IL-6 and IL-1β via the MAPK and the NF-κB signaling pathways [128]. After binding to its receptor, TNFR1, TNF-α creates a receptor signaling complex named complex I [193]. Complex I favors the activation of genes for TNF-α and IL-6 [194,195]. TNF-α can also create complex II (also named “cell death complex”) that can induce apoptosis through the activation of caspase-3 [194,196,197]. 

In obesity, IL-1β is produced mainly after TLR4-mediated FFA activation of canonical inflammasomes, which is associated with increased expression of inflammation [194,198]. The adipocyte and macrophage production of IL-1β within the WAT contributes to the inflammation process and plays a role in the adipocyte–neutrophil interplay [199]. After its release, IL-1β contributes to vascular endothelial cell damage and other cardiovascular complications of diabetes [200,201,202,203,204,205,206,207]. The endothelial dysfunction may be modulated by local factors, vascular morphometry, diet, exercise, and other patient-related factors [208,209,210,211].

IL-6 is released by adipocytes, fibroblasts, and endothelial cells and stimulates the production of CRP in the liver [168]. The plasmatic concentrations correlate with patient body weight (i.e., waist size, BMI) but also with FFA levels [212,213].

CRP acts as a marker of systemic inflammation and its levels show high sensitivity in obesity [128]. 

As previously mentioned, MCP-1 is responsible for macrophage recruitment and proliferation in obese AT [214,215]. MCP-1 is released by macrophages and endothelial cells and shows increased levels in obesity [216]. 

Another pro-inflammatory CK, IL-18, is activated through the NLRP1 inflammasome, increases the expression of additional pro-inflammatory CKs, and, overall, amplifies the inflammation associated with obesity [29,217].

### 5.3. Obesity and Adipokines

**Figure 3:**
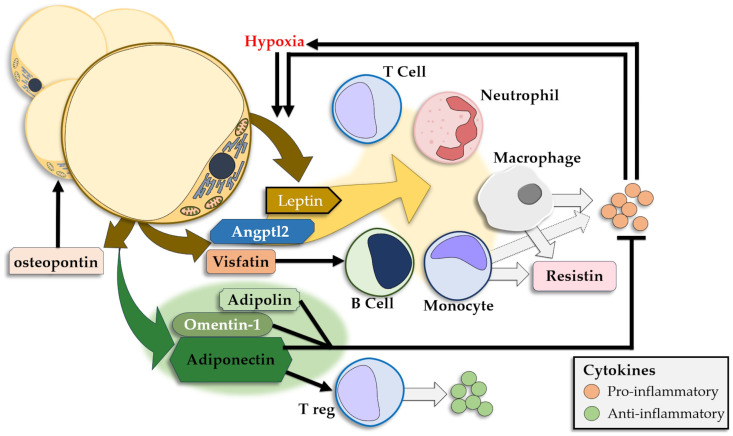
Adipokines released by the adipose tissue and their pro-/anti-inflammatory effects.

The WAT releases a large number of adipokines that either show anti-inflammatory effects or promote inflammation, and releases insulin resistance-inducing CKs [218,219] (Figure 3). Leptin and adiponectin are the most prominent adipokines and show significantly different plasmatic concentrations in obese individuals, with high levels of leptin and low levels of adiponectin [176,220]. Other adipokines modulate the signaling pathways in the AT as well as the cellular and CK interplay in local and systemic inflammation.

Leptin regulates food intake and energy consumption, therefore playing a major role in the management of body weight [221,222]. With the development of fatty tissue, circulating levels of leptin increase [128]. Moreover, leptin is produced in significantly higher quantities in the SAT compared to the VAT due to a direct correlation with adipocyte size [223,224,225]. When circulating levels of leptin are reduced, the hypothalamus regains its sensitivity to leptin and subsequent limitation of weight gain ensues, alongside an increase in the sensitivity to insulin [226]. Leptin is part of a positive feedback loop, where, acting as a pro-inflammatory molecule, adipokine leads to increased expression of inflammatory mediators and hypoxia, leading to subsequent leptin expression in the AT [227,228,229,230]. 

Through its receptors, leptin modulates the activity of monocytes, macrophages, T and B cells, and neutrophils [231,232]. Following leptin stimulation, B cells stimulate monocyte proliferation, while T cells will show decreased apoptosis and increased proliferation and activity with polarization toward Th1 cells [215,233,234,235,236]. A concurrent increase in levels of pro-inflammatory CKs such as TNFα, IFN-γ, IL-2, IL-6, IL-10, and IL-12 and a corresponding decrease in levels of IL-4 and IL-10 is observed [234,237]. Furthermore, in neutrophils, leptin leads to enhanced ROS production, proliferation, and migration [238,239,240].

The leptin/adiponectin (L/A) ratio was proposed as a predictor of cardiometabolic events due to its identified correlation with metabolic syndrome, IR, and also with carotid intima media thickness [241,242,243,244]. A study on adolescents concluded that the L/A ratio is more accurate than adiponectin alone in identifying the risk of IR [244]. Moreover, the adiponectin/leptin (A/L) ratio is inversely related to the BMI [245] and to serum inflammatory markers such as CRP and serum amyloid A. An A/L ratio over 1 is considered normal, between 0.5 and 1 it indicates a moderate cardiovascular risk, and below 0.5 it is linked to a severe cardiovascular risk [246,247]. The A/L ratio increases with exercise due to the increase in adiponectin and concurrent decrease in leptin levels [248], and increases in diets associated with decreasing leptin levels such as fish or omega-3 polyunsaturated fatty acids supplements, or by fiber ingestion, accompanied by increases in adiponectin levels in all above-mentioned cases [249,250]. Statins also increase the A/L ratio, as shown in experimental studies [251]. The L/R ratio positively correlates with PCR levels and HOMA-IR, regardless of the presence of obesity or other metabolic risk factors in non-diabetic patients [252]. However, in obese patients, an inverse correlation between the L/A values and serum levels of soluble P-selectin was found [253,254]. Some authors consider that adiponectin and leptin, and consequently the L/A ratio, represent more accurate biomarkers for atherosclerosis than the traditional risk factors such as dyslipidemia, diabetes, or arterial hypertension [255,256].

Resistin is another adipokine with increased levels in obesity, albeit with a low correlation between plasma levels and the BMI or insulin resistance [168,257,258]. While mainly produced in monocytes and macrophages, resistin can also be produced in adipocytes, but only in mice [259]. The effects of resistin are an increase in the pro-inflammatory response in adipocytes and a TLR4-mediated release of pro-inflammatory CKs such as IL-1β, IL-2, and IL-6 from white blood cells [260].

Angiopoietin-like protein 2 is produced by adipocytes, with higher levels in obesity, and regulates the cellular inflammatory response in the AT, mainly acting on monocytes and macrophages [261].

Visfatin is an adipocyte-produced growth factor for B-cell precursors and presents effects similar to insulin [262]. Its values increase in obesity, apparently reflecting the regulatory response to increased glycemia; however, highly increased levels reflect an increase in inflammation that may correlate with the development of type 2 diabetes, IR, and other metabolic dysfunctions [263].

Calprotectin also presents increased expression in obesity and regulates WAT inflammation by contributing to macrophage recruitment and monocyte infiltration [129,264].

Chemerin is another adipokine involved in inflammation and also plays a role in angiogenesis and adipogenesis [265]. The levels of chemerin correlate with the BMI, insulin resistance, and the risk of developing type 2 DM, as well as with liver markers of inflammation [265,266,267,268].

A pro-inflammatory adipokine, osteopontin, is expressed in adipocytes, macrophages, endothelial cells, lymphocytes, and other cells, and contributes to adipogenesis [222].

Released mainly by adipocytes, adiponectin shows decreased levels in obesity and AT inflammation [128,269]. With the most potent anti-inflammatory effect, adiponectin inhibits the release of pro-inflammatory CKs, like TNF-α and IL-6, while stimulating IL-10 release [128,270]. Adiponectin polarizes macrophages towards the M2 type, and decreases ROS production, monocyte adhesion, and endothelial cell activation [271,272,273,274].

Secreted frizzled-related protein 5 correlates inversely with pro-inflammatory CK production and macrophage expression in the AT [128].

While produced specifically by adipocytes, adipolin is downregulated in obesity and promotes anti-inflammatory CKs while inhibiting the pro-inflammatory response and macrophage accumulation [128,275].

Omentin-1 is a strong anti-inflammatory, anti-oxidative adipokine produced in the stromal cells of the VAT [276]. It shows decreased expression and inverse correlation to the BMI and the waist size in obesity [222].

## 6. Regulation of AT Expansion and Interplay with Inflammation 

The expansion of the AT during excessive caloric intake occurs either through the accumulation of triglycerides in adipocytes leading to hypertrophy or through the increase in differentiation of PAs into adipocytes, leading to hyperplasia. Apoptosis and autophagy regulate and limit the adipocyte expansion; however, when adipocytes are saturated with lipids, ectopic storage of fat can occur, with deposition of lipids in various regions outside the AT [277]. 

As previously mentioned, adipocyte hypertrophy is accompanied by an increased expression of MCP-1 leading to amplified macrophage recruitment and clustering in CLSs [235,278]. As a result, an NF-κB-mediated TNF-α secretion occurs alongside a release of macrophage-activating triglycerides [105,278,279]. Subsequently, insulin resistance develops via the downregulation of the IRS-1 pathway, leading to impaired glucose absorption into the AT [45,97,280,281,282].

With hypertrophy, an important adipocyte organoid and cell membrane dysfunction occurs, leading to an increased predisposition to apoptosis, inflammation, and hypoxia [283]. 

The hypertrophic adipocytes are skewed towards pro-inflammatory effects, with increased expression of CKs such as TNF-α and IL-6, while anti-inflammatory CKs and adipokines are downregulated [33,34,35]. Conversely, during hyperplasia, the smaller adipocytes contribute to a smaller degree to inflammation [284]. 

Furthermore, there is a fundamental relationship between inflammation and cardiometabolic obesity-related complications. Metabolically healthy obesity (MHO) is described in obese people who do not exhibit cardiometabolic abnormalities [285]. Interestingly, MHO is characterized by lower adipocyte size and lower infiltration of immune cells into fat deposits, as well as altered dynamics of adipokine secretion with lower levels of CRP but higher levels of neuroregulin and adiponectin [35,99,169].

One of the hallmarks of AT expansion is angiogenesis and both processes seem to negatively correlate with inflammation in MHO [11,286,287,288]. However, the development of hypoxia modulates the secretion of adipokines and increases the hypoxia-inducible factor 1a (HIF-1a) expression and, subsequently, the recruitment of monocytes into the AT [20,289,290,291]. The interplay between angiogenesis and hypoxia is extremely relevant due to the fact that the AT expansion limits vascular supply while increasing oxygen consumption, leading to hypoxia [99,289,290,292]. 

AT expansion is opposed by apoptosis and autophagy. Apoptosis in the WAT is mediated by caspase 8 and is a crucial part of the inflammatory cascade and development of insulin resistance in obesity [121,293]. Adipocyte apoptosis contributes to macrophage recruitment and infiltration in the AT and favors polarization towards type 2 macrophages which carry anti-inflammatory effects [194,294]. On the other hand, autophagy acts as a physiological limitation to exaggerated hyperplasia/hypertrophy and restricts the development of inflammatory processes [295]. 

## 7. Managing Weight Loss in Obesity-Associated Inflammation 

The development of adipose tissue inflammation triggers subsequent cardiovascular events and therefore represents a major reason for the treatment of obesity. In this regard, with weight loss, fewer cardiovascular events and less inflammation are to be expected. However, an experimental study showed controversial results, i.e., a phasic response to weight loss; in the beginning, lipolysis led to an increase in ATMs with sustained inflammation, and only in the later stages did the number of ATMs decrease, with gradual extinguishing of the inflammatory processes [296]. Some studies on formerly obese people showed that inflammation in the AT may persist long after weight loss, which is in contrast to the inflammatory processes in the liver, for instance, where the inflammation and lipid storage decline immediately after weight loss [141]. 

Weight loss triggers early changes to the macrophage phenotypes, with a shift from the lipid-binding population that is prevalent in the obese AT to the phagocytosis-type population [297].

As mentioned above, attenuation of the WAT inflammation in obese individuals was expected to lead to corresponding decreases in cardiovascular risks and complications. However, recent research has reported mixed results.

A moderate weight loss, of 3 to 10% of body weight showed some health improvements; however, at least 10% is required for sustained clinically meaningful effects [298,299,300]. The arsenal for weight loss relies on a combination of caloric deficit nutrition and physical exercises; failure to attain optimal weight may be followed by pharmaceutical treatment, which can include one of the approved medications for clinical use [300,301,302]. Surgical interventions for weight loss are another viable option, especially considering that a significant proportion of individuals who have achieved the ideal weight through a diet and physical activity, alone or in combination, are unable to sustain their weight loss [303].

### 7.1. Dietary Interventions

The weight loss through diet is accompanied by an increased expression of genes stimulating adipogenesis, demonstrating that PA signaling and secretion are modulated by weight loss [304]. In dietary interventions applied to obese subjects, an evident decrease in obesity-related inflammatory markers (ORIMs) occurs when patients lose over 10% of their body mass, regardless of the type of diet [305,306,307,308].

### 7.2. Physical Activity

Physical activity contributes to a smaller extent to weight loss compared with dietary interventions; this phenomenon occurs due to the increase in fat-free mass [309]. Additionally, the impact on the ORIMs is lower in physical activity alone.

### 7.3. Combination of Diet and Physical Activity

Similarly to dietary interventions alone, at least a 10% body weight decrease is needed to observe significant decreases in the levels of ORIM [310,311,312]. Physical exercise with high intensity appears to favor macrophage polarization towards type 2, therefore leading to an anti-inflammatory dominance within the AT [270,313]. As the reduction in inflammation occurs with latency after weight loss, a sustained decrease in body mass is essential to obtain a favorable decrease in ORIM levels [314,315,316].

### 7.4. Surgical Interventions

In obesity, a variety of bariatric surgical procedures are available, such as fat removal, gastric restrictive surgery, gastric reduction, or gastric bypass. While the adipocyte number and mass are reduced through bariatric surgery, inflammation attenuation does not always occur. This is demonstrated by abdominal liposuction, a procedure that eliminates high quantities of SAT but is not accompanied by corresponding decreases in the levels of ORIMs [317]. Various procedures and techniques have been employed in bariatric surgery, involving robotic, needlescopic, and laparoscopic approaches, some adapted and extended from other fields [318,319,320,321,322,323]. Most surgical procedures are followed by a reduction in CRP and leptin, with increases in adiponectin levels. While decreases in CRP levels are reported after 3 months by numerous studies, TNF-α and IL-6 do not exhibit similar tendencies [324,325].

Bariatric surgery leads to a decrease in both pro-inflammatory macrophages and total macrophages and can also polarize the macrophage phenotype towards anti-inflammatory [326,327,328].

Neutrophil levels are also decreased after bariatric surgery-associated weight loss [329]. Moreover, decreases in CD4+ and CD8+ circulating T-cell levels are recorded after gastric sleeve in morbidly obese patients [330]. In patients with DM, gastric banding leads to a decrease in Th1 levels with no effects on the Th2 population [331,332]. ROS generation also diminishes after bariatric surgery-induced weight loss [328]. Additionally, gastric bypass leads to a decreased expression of pro-inflammatory genes [333].

Bariatric surgery also influences the ECM composition of the AT. Obesity is associated with increased collagen gene expression in the WAT which leads to high ORIM levels, increased inflammation, and insulin resistance [173,334,335,336,337]. The evaluation of genetic markers of the AT in obese patients undergoing bariatric surgery showed that lytic enzymes are expressed in larger quantities in obesity, leading to the breakdown of the ECM, a phenomenon that may be alleviated several months after bariatric surgery [45,338]. 

Blocking or antagonizing the effects of pro-inflammatory CKs does not seem to be an effective way to counter the inflammation in obesity. Drugs inhibiting TNF-α do not seem effective, most likely due to low penetrance into the AT [20]. Conversely, anti-IL-1β antibodies show anti-inflammatory effects and can lead to a decrease in cardiovascular events, showing good promise in the management of atherosclerotic disease [339]. 

Nuclear factor erythroid2 p45-related factor 2 has a significant anti-inflammatory, anti-oxidative role, however, attempts to increase its activation did not yield the expected results [10,340,341,342,343]. 

In some cases, rapid weight loss via bariatric surgery or extreme caloric restriction may have detrimental effects, such as the paradoxical increase in pro-inflammatory CKs [344,345]. However, among all the therapeutic methods described here, low-calorie dieting and gastric bypass surgery led to the most effective weight loss with the highest drops in ORIM levels [308].

## 8. Conclusions

The interplay between obesity and inflammation is complex and involves a variety of cellular and humoral factors. Macrophages are the main cellular component involved in the chronic inflammation of the obese adipose tissue and their modulation is instrumental in the interaction between the inflammatory and immune responses and can be a key factor in the medical intervention in obese patients. The development of adipose tissue inflammation triggers subsequent cardiovascular events and therefore represents a major reason for the treatment of obesity. There are a variety of options in the management of weight loss; however, it should be noted that due to the complexity and particular features of the adipose tissue, the inflammation within may persist for long periods of time, compared to the inflammatory processes in the liver, for instance, where the inflammation and lipid storage decline immediately after weight loss. Understanding the pathophysiological changes in the adipose tissue and the interplay with chronic inflammation can assist in the design of future studies and reveal opportunities for the development of more efficient therapies for obesity.

## Data Availability

No new data were created or analyzed in this study. Data sharing is not applicable to this article.
